# Kinetics of the thermal inactivation and the refolding of bacterial luciferases in *Bacillus subtilis* and in *Escherichia coli* differ

**DOI:** 10.1371/journal.pone.0226576

**Published:** 2019-12-23

**Authors:** Eugeny Gnuchikh, Ancha Baranova, Vera Schukina, Ilyas Khaliullin, Gennady Zavilgelsky, Ilya Manukhov

**Affiliations:** 1 Moscow Institute of Physics and Technology, Dolgoprudny, Moscow Region, Russia; 2 National Research Center, Kurchatov Institute, GOSNIIGENETIKA, Moscow, Russia; 3 School of Systems Biology, George Mason University, Fairfax, VA, United States of America; 4 Research Centre for Medical Genetics, Moscow, Russia; Russian Academy of Medical Sciences, RUSSIAN FEDERATION

## Abstract

Here we present a study of the thermal inactivation and the refolding of the proteins in Gram positive *Bacillus subtilis*. To enable use of bacterial luciferases as the models for protein thermal inactivation and refolding in *B*. *subtilis* cells, we developed a variety of bright luminescent *B*. *subtilis* strains which express *luxAB* genes encoding luciferases of differing thermolability. The kinetics of the thermal inactivation and the refolding of luciferases from *Photorhabdus luminescens* and *Photobacterium leiognathi* were compared in Gram negative and Gram positive bacteria. In *B*. *subtilis* cells, these luciferases are substantially more thermostable than in *Escherichia coli*. Thermal inactivation of the thermostable luciferase *P*. *luminescens* in *B*. *subtilis* at 48.5°С behaves as a first-order reaction. In *E*.*coli*, the first order rate constant (K_t_) of the thermal inactivation of luciferase in *E*. *coli* exceeds that observed in *B*. *subtilis* cells 2.9 times. Incubation time dependence curves for the thermal inactivation of the thermolabile luciferase of *P*. *leiognathi* luciferase in the cells of *E*. *coli* and *B*. *subtilis* may be described by first and third order kinetics, respectively. Here we shown that the levels and the rates of refolding of thermally inactivated luciferases in *B*. *subtilis* cells are substantially lower that that observed in *E*. *coli*. In *dnaK*-negative strains of *B*. *subtilis*, both the rates of thermal inactivation and the efficiency of refolding are similar to that observed in wild-type strains. These experiments point that the role that DnaKJE plays in thermostability of luciferases may be limited to bacterial species resembling *E*. *coli*.

## Introduction

When exposed to mildly elevated temperatures, eukaryotic and prokaryotic thermolabile proteins transiently undergo partial or complete unfolding, resulting in a loss of their activity [1]. Persistence of the heat stress prevents the proteins from refolding to their native state, while favoring alternative, beta-sheet enriched conformations. To prevent misfolding, eukaryotic and prokaryotic cells employ a variety of molecular chaperones, the most abundant and best studied of which being Hsp60-Hsp10 (GroEL-GroES), Hsp70-Hsp40-nucleotide exchange factor (DnaK-DnaJ-GrpE), Hsp100 (ClpA–ClpB–ClpX) and so-called small chaperones sHsp (IbpA-IbpB) [2–10].

In studies of the folding, misfolding and refolding conditions in *Escherichia coli*, bacterial and firefly derived luciferases often serve as model substrates [10–13]. Moreover, luminescent Gram positives bacteria have their use as biosensors suitable for clinical applications [14–18]. Despite enormous biotechnological importance of Gram positive cells in general, and of *B*. *subtilis*, a workhorse of industrial recombinant protein production in particular, the mechanisms of the folding and the refolding of the proteins in these bacterial cells remain enigmatic.

In present work we use the model bacterial luciferases differing in their thermostability to investigate the thermal inactivation and the refolding of the proteins in Gram positive *B*. *subtilis*. A variety of bright luminescent *B*. *subtilis* strains which express *luxAB* genes encoding luciferases from bacteria *P*. *luminesce*ns [14] and *P*. *leiognathi* [15], are utilized in the comparative study of the kinetics of the thermal inactivation and the refolding of the luciferases in Gram negative and Gram positive bacteria. In particular, we evaluated effects of *dnaKJ* genes on luciferase thermostability in cellular environments of *B*. *subtilis* and *E*. *coli*.

## Materials and methods

### Bacterial strains, plasmids, and growth conditions

Bacterial strains are presented in Table 1. Plasmids are presented in Table 2.

**Table 1:** Bacterial strains.

Strain	Genotype	Source
*E*. *coli* K12 BW25113	*lacI*^q^ *rrnB*_T14_ Δ*lacZ*_WJ16_ *hsdR514* Δ*araBAD*_AH33_ Δ*rhaBAD*_LD78_	obtained from Keio Collection
*E*. *coli* K12 *JW0013*	Derivative of BW25113 *ΔdnaK*::*kan*	obtained from Keio Collection
*B*. *subtilis* 168	*trpC2*	obtained from VKPM
*B*. *subtilis NBS 2001*	Derivative of 168, *ΔdnaK-dnaJ*::*Spc*^*r*^	obtained from H. Yoshikawa. [19].

**Table 2:** Plasmids used in the work.

Plasmid	Description	Source
pMWAL-1TPpur	pMWAL-1 [20] based shuttle vector made by cloning of the promoter Ppur from *Bacillus amyloliquefaciens*. Includes pMW118 and pBS72 replicons along with chloramphenicol (Cm^r^) and ampicillin (Ap^r^) resistance genes.	Obtained from V.A. Lifshits (GosNIIgenetika)
pXen5	Integrative shuttle vector with two replicons: pE194 for *B*. *subtilis* and pMB9 oriC for *E*. *coli*, a lux-operon cassette ABCDE lux from *P*. *luminescens* with modified SD sequences, transposase and IR repeats as well erythromycin (Erm^r^) and kanamycin (Km^r^) resistance genes.	[14]
pLF22ABleo	Includes α and β subunits of *P*. *leiognathi* luciferase expressed from promoter P*rep* in plasmid pLF22 (Cm^r^).	[15]
pMWAL-1Ppur-dhfr-t1t2-MCS (изменить название)	pMWAL-1TPpur–based shuttle vector made by the cloning of multiple cloning site sequence and dihydrofolate reductase encoding gene which confers resistance to trimethoprim (Tp^r^). Plasmid also contains Cm^r^ and Ap^r^.	Present work
pPfbaA-MCS	pMWAL-1Ppur_dhfr_t1t2_MCS based shuttle vector made by adding promoter of fructose-1,6-bisphosphate aldolase gene (P*fbaA*). (Tp^r^, Cm^r^ and Ap^r^).	Present work
pPfbaA-XenAB	Plasmid resulting from the cloning of α and β subunits of the luciferase from *P*. *luminescens* into pPfbaA_MCS (Tp^r^, Cm^r^ and Ap^r^).	Present work
pPfbaA_LeoAB	Plasmid resulting from the cloning of α and β subunits of the luciferase from *P*. *leiognathi* into pPfbaA_MCS (Tp^r^, Cm^r^ and Ap^r^).	Present work
pTZ57R-xenAB	Plasmid resulting from the cloning of α and β subunits of the luciferase from *P*. *luminescens* under promoter *Plac* into vector pTZ57R (Ap^r^).	Present work
pTZ57R-leoAB	As pTZ57R-xenAB but *lux* gene of luciferase take from *P*. *leiognathi*.	Present work

*E*. *coli* and *B*. *subtilis* was grown either in LB media, with constant aeration at 200 rpm at 37°С unless indicated otherwise. Solid media plates were prepared using 1.5% of agar.

For selection, media we made with spectinomycin 150 μg/ml, ampicillin 100 μg/ml and chloramphenicol 10 μg/ml.

### Transformation

*B*. *subtilis* cells were transformed according to the protocol of Spizizen [21]. *E*. *coli* cells were transformed using calcium chloride protocol [22].

### Enzymes and chemical substances

The substrate for luciferase *n*-decanal was from Sigma-Aldrich (USA). Enzymes for cloning were purchased in Promega (USA). Media were from Helicon (Moscow, Russia). Oligonucleotides were made by Syntol (Moscow, Russia).

### Constructing the plasmids

Primers utilized for constructing the plasmids are described in Table 3. As a backbone for assembly of biosensors we selected shuttle plasmid pMWAL-1TPpur with two origins pMW118 (GenBank: AB005475) and pBS72 [23], which allows teta-type replication, as well as amplicillin and chloramphenicol resistance gens *bla* and *cat* for *E*. *coli* and *B*. *subtilis*, respectively. With an aid of P1/P2 primers, trimethoprim resistance gene *dhfr* from *B*. *cereus* ATCC14579 was introduced into the plasmid pMWAL-1TPpur under its P*pur* promoter. With an aid of P3/P4 primers, *rrnB* terminator T1T2 was introduced to build a promoterless plasmid pMWAL-1Ppur_dhfr_t1t2_MCS. In turn, with an aid of primers P5/P6, this construct was modified by inserting constitutive promoter of fructose-1,6-bisphosphate aldolase gene (P*fbaA*) [24] into SacI recognition site, thus, resulting in plasmid pPfbaA_MCS. Later, plasmid pPfbaA_MCS was utilized for cloning of α and β subunits of luciferases from *P*. *luminescens* and *P*. *leiognathi*, which were amplified on the pXen5 [14] and pLF22ABleo [15] templates using P7/P8 and P9/P10 primers, respectively, to obtain pPfbaA_XenAB and pPfbaA_LeoAB plasmids, respectively. Both subunits of *P*. *luminescens* and *P*. *leiognathi* luciferases were cloned into pTZ57R vector to obtain constructs pTZ57R_xenAB (with pair of primers P7/P8) and pTZ57R_leoAB (with pair of primers P9/P10), respectively.

**Table 3:** Primers utilized for constructing the plasmids.

P1	5-GTTTCTACCCGGCTGCCGTAATAAAGGAGGTTTACCGATGATTGTTTCATTTATGGTCGCTATG-3
P2	5-TCGGTACCCGGGGATCCTCGATTCTCCTCCTCTTTCTATATTAGT-3
P3	5-GTACTAATATAGAAAGAGGAGGAGAATCGAGCTGATGCAAAAACGAGGCTAGTTTAC-3
P4	5-CGGCCGCTCGAGGGGCCCGGCGCGCCGGATCCCCATGCCGAACTCAGAAGTGAA-3
P5	5-GCCGCGGTACCGAGCTTTTTCTCCATAACTAGGATACCAAC-3
P6	5-AAAGAAGAGCTTTCAGGtATTCGAATCATGTCATTATGTTGCCGATTTG-3
P7	5-ACCGCGGCCGCTCGAGGAAGCAAGAGGAGGACTCTCTATG-3
P8	5-GCCGGGCCCCTCGAGATTTCAACCTGGCCGTTAATAATGAATGA-3
P9	5- ATCGCGGCCGCTCTCAGATCGGAAGGTGGAAGAA -3
P10	5- TCGGGGCCCGTACCTCGCGAATGCATCTA-3

### Measurement of the intensity of bioluminescence

Cell were prepared by overnight cultivation at 30°С with aeration at 200 rpm in LB media with chloramphenicol or ampicillin, then diluted 1:100 in LB media with chloramphenicol, grown till reaching *OD* = 0.4–0.5, then incubated at a certain temperature. To eliminate *de novo* protein synthesis, incubation media was supplemented with following antibiotics: chloramphenicol (167 μg/ml) for *E*. *coli* and tetracycline (60 μg/ml) for *B*. *subtilis*.

To measure the intensity of bioluminescence the cells were sampled into the 200-μl test tubes like in [25]. Two μl of 0.1% *n*-decanal dissolved in ethanol were added to final concentration of 0.001%. Cells are placed in the luminometer without shaking at room temperature (~20°С), with direct measurements of total bioluminescence (in RLU, relative light units) using “Biotox-7” (LLC EKON, Russia). In five seconds timeframe, luciferase substrate *n*-decanal enters the cells and participates in light producing reaction.

## Results

Hybrid plasmid with P_*fbaA*_ or P_*lac*_ controlled *luxAB*-genes were introduced to *B*. *subtilis* and *E*. *coli* cells, respectively. The levels of resultant strain bioluminescence in cultures sampled at varying *OD* are shown in Table 4. As could be seen form Table 4, bioluminescence intensities observed for *B*. *subtilis* and for *E*. *coli* cultures are approximately the same.

**Table 4:** A comparison of the levels of bioluminescence observed in *B*. *subtilis* 168 and *E*. *coli* cells transformed by plasmids with *luxAB*-genes.

Strains*	*OD* = 0.1	*OD* = 0.2	*OD* = 0.5	*OD* = 1,0
B.s. pFba-leoAB	4500±600**	12000±1000	100000±15000	300000±50000
B.s. pFba-xenAB	3000±500	10000±1000	45000±6000	110000±15000
E.c. pTZ57R-leoAB	12000±2000	28000±3300	75000±7000	160000±45000
E.c. pTZ57R-xenAB	10000±1250	19000±2050	41000±5200	90000±17000

* B.s.—*B*. *subtilis* 168; E.c.—*E*. *coli* K12 BW25113

** Levels of luminescence are shown in RLU, relative light units, with background luminescence levels at 50 RLUs.

To quantify relative thermostability of luciferases *in vivo*, the *luxAB* gene expressing cells of *B*. *subtilis* 168 and *E*. *coli* BW25113 were grown at 28°С and 37°С for cells with luciferase from *P*. *leiognathi* and *P*. *luminescens*, respectively, until *OD* = 0.4–0.6 was reached. Then the tetracycline or chloramphenicol was added, and incubation was continued at elevated temperatures. In this experiment, addition of antibiotics prevented protein synthesis *de novo*.

**Fig 1:**
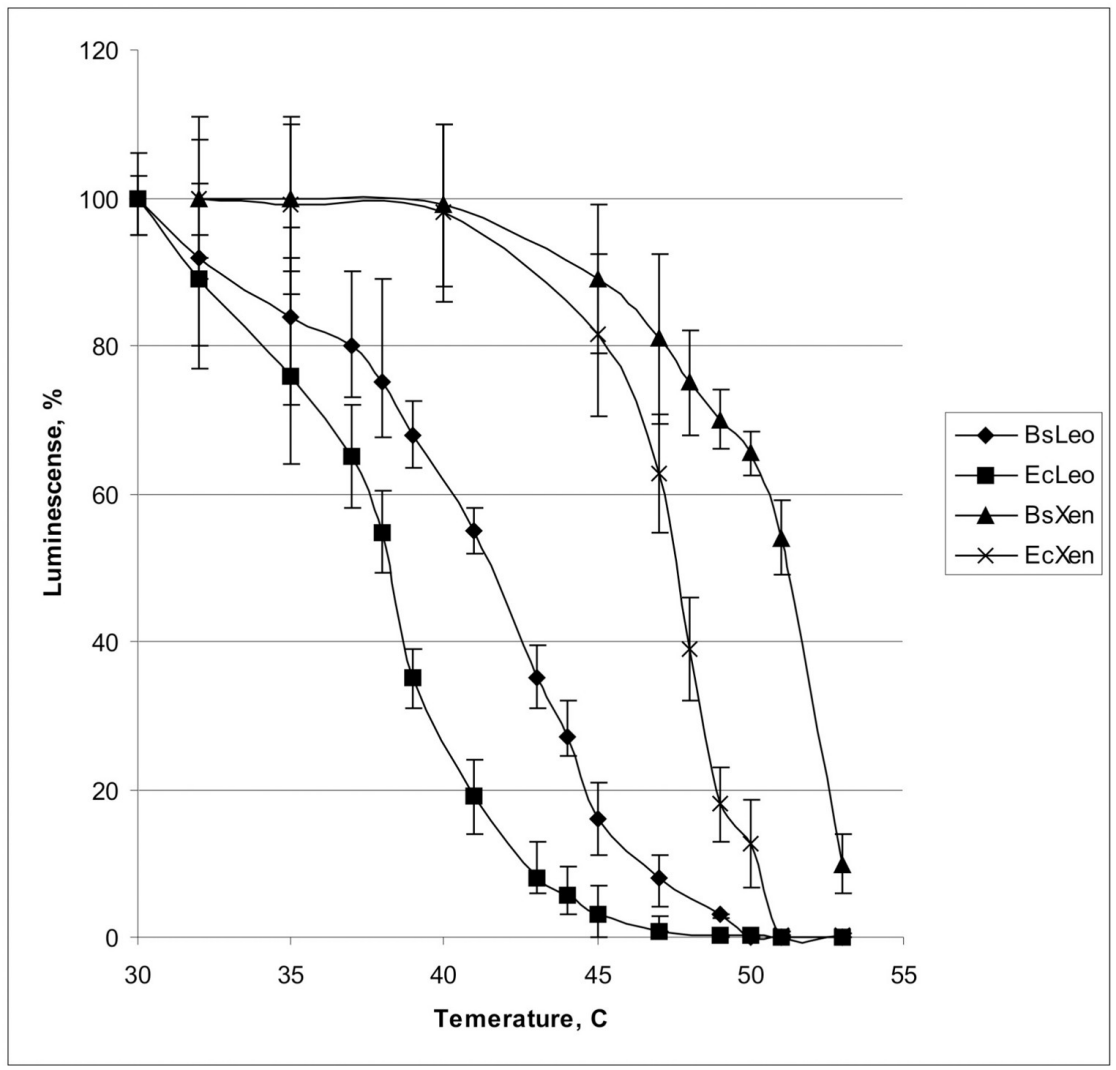
A plot reflecting relative drops in the levels of bioluminescence during 15 minutes of incubation at various temperatures. BsLeo—*B*. *subtilis* 168 (pFba-leoAB). EcLeo- *E*. *coli* BW25113 (pTZ57R_leoAB). BsXen—*B*. *subtilis* 168 (pFba-xenAB). EcXen—*E*. *coli* BW25113 (pTZ57R_xenAB).

Fig 1 shows the luminescence of *B*. *subtilis* 168 (with pFba-xenAB or pFba-leoAB) and *E*. *coli* BW25113 cells (with pTZ57R-xenAB or pTZ57R-leoAB) at various temperatures.

As could be seen at Fig 1, in *B*. *subtilis* the luciferases display higher thermostability than in *E*. *coli*. When expressed in *B*. *subtilis*, each luciferase reached inactivated state at the temperature of 3–5°С higher than in *E*. *coli*. In course of subsequent experimentation with thermal inactivation of luciferase in *B*. *subtilis* and *E*.*coli* cells *in vivo*, the differences in luciferase thermostabilities were taken into account.

According to data obtained *in vitro* [26] and *in vivo* in *E*. *coli* cells [11], *P*. *leiognathi* luciferase is significantly more thermolabile than luciferase from *P*. *luminescense*. The data in Fig 1 show that the same difference persists in the cells of *B*. *subtilis*.

**Fig 2:**
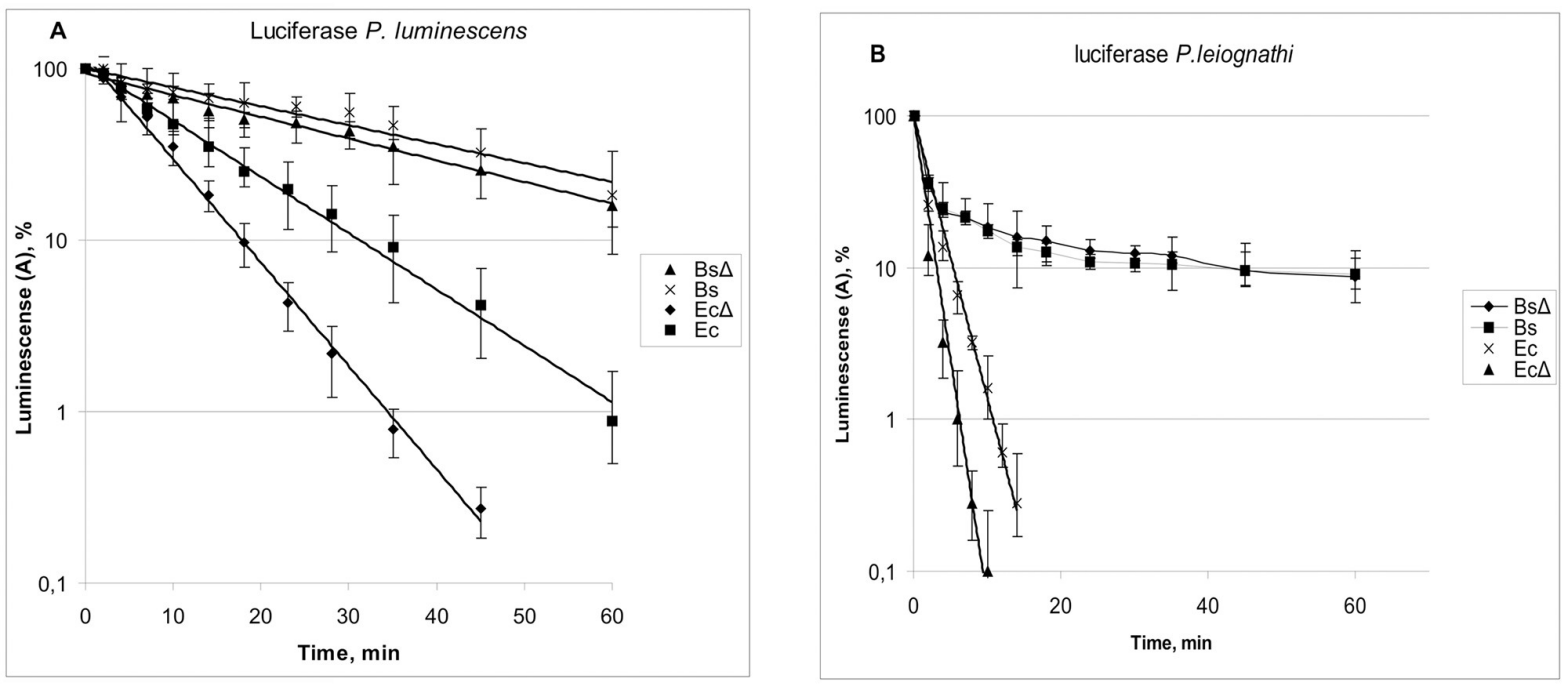
Plots of relative luminescence of *B*. *subtilis* and *E*. *coli* cells expressing luciferases *P*. *luminescens* (A) and *P*. *leiognathi* (B), and exposed to 48,5°С and 41°С, respectively. Приведены средние значения шести экспериментов.Bc—*B*. *subtilis* 168; BcΔ—*B*. *subtilis* NBS2001 Δ*dnaKJ*::*spc*; Ec–*E*. *coli* BW25113; EcΔ—*E*. *coli JW0013 ΔdnaK*::*kan*;.

Kinetics of luciferase thermal inactivation *in vivo* in *B*. *subtilis* cells were compared to that observed in cells of *E*. *coli*. Fig 2 presents inactivation kinetics at 41°С or 48,5°С for luciferases from *P*. *leiognathi* (Fig 2A), and *P*. *luminescens* (Fig 2B), respectively.

As could be seen from the data presented at Fig 2A, in all strains the bioluminescence intensity drops observed for *P*. *luminescens* luciferase are well described by a semi-logarithmic graph (lg A–time), and, therefore, thermal inactivation of this luciferasae is a first-order reaction. Table 5 shows respecitive first order rate constants (Kt).

**Table 5:** First order rate constants (Kt) for *P*. *luminescens* luciferase inactivation at 48.5°С.

Strain	Rate constant, K_t_, min^-1^	Correlation coefficient, R^2^
*B*. *subtilis*	0.0254	0.957
*B*. *subtilis ΔdnaKJ*	0.031	0.976
*E*. *coli*	0.074	0.992
*E*. *coli ΔdnaK*	0.132	0.993

As could be seen from Table 5, for luciferase of *P*. *luminescens* the ratio of the rate constants for wild type strains of *E*. *coli* and *B*. *subtilis was 2*.*9*, while for Δ*dnaK* mutant strains of same bacteria this ratio was 4.3.

**Fig 3:**
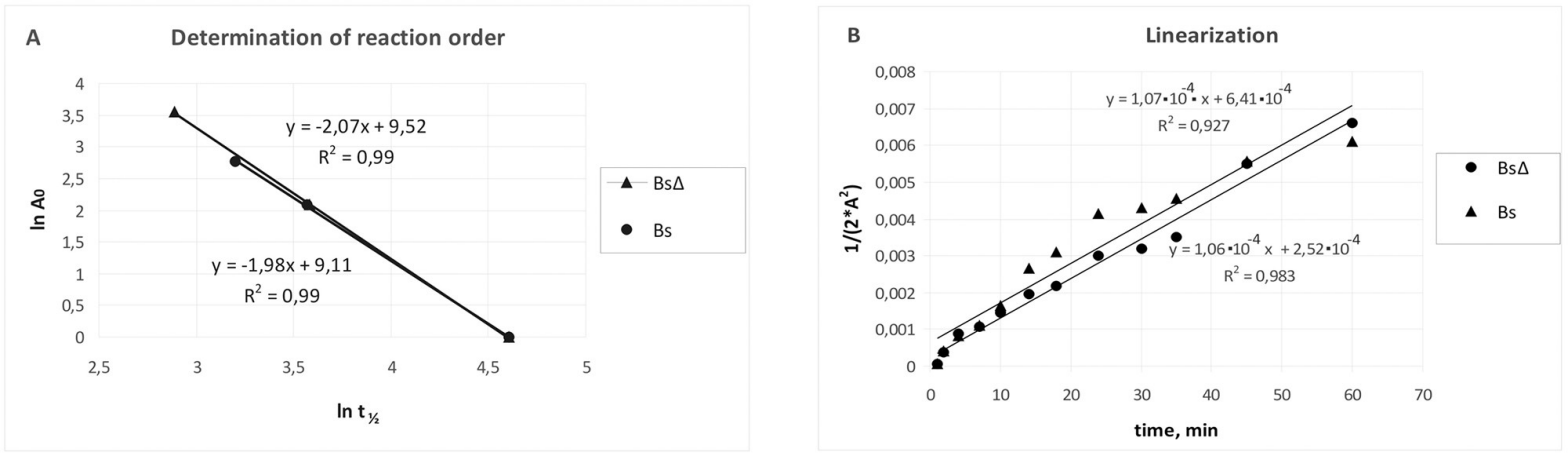
Dependence of initial bioluminescence and the time to semi-inactivation at 41°С (A) and linearization of data describing inactivation in coordinates ½*A^-2^ *Vs*. *t* (B) for *B*. *subtilis* cells expressing luciferase *P*. *leiognathi*. Bc—*B*. *subtilis* 168; BcΔ—*B*. *subtilis* NBS2001 Δ*dnaKJ*::*spc*; A–units of activity.

As could be seen from the data presented at Fig 2B, for luciferase of *P*. *leiognathi* expressed in *E*. *coli* cells the bioluminescence intensity drops are also well described by a semi-logarithmic graph (lg A–time), while in *B*. *subtilis* cells respective kinetics are substantially more complex. To find out the order of this reaction, Rakovsky techniques was employed [27, §301 p. 466] by linearizing it in coordinates ln *t*_1/2_ *Vs*. ln *A*_0_, where *A*_0_ –initial activity, *t*_1/2_ –time to semi-inactivation (Fig 3A).

As could be seen from Fig 3A, for both lines the slope is close to 2, indicating that the kinetics of this reaction is of a third-order. These data were linearized in coordinates ½*A^-2^ *Vs*. *t* and approximated by lines shown on Fig 3B. Table 6 shows rate constants for *P*. *leiognathi* thermal inactivation at 41°С in *E*. *coli* and *B*. subtilis cells.

**Table 6:** Rate constants (K_t_) of *P*. *leiognathi* luciferase thermal inactivation.

Strain	Order of the reaction	Rate constant (K_t_)	Correlation coefficient, R^2^
*E*. *coli*	1	0,427 min^-1^	0,986
*E*. *coli ΔdnaK*	1	0,735 min^-1^	0,972
*B*. *subtilis*	3	1,06•10^−4^•min^-1^•A^-2^*	0,983
*B*. *subtilis ΔdnaKJ*	3	1,07•10^−4^•min^-1^•A^-2^	0,927

* A -–units of activity

Notably, the difference in the rates of *P*. *leiognathi* luciferase thermal inactivation at 41°С in the cells of *E*. *coli* and *B*. *subtilis*, which are described by first order and third order kinetics, respectively, leads to a prominent, orders-of magnitude difference in the levels of cell bioluminescence at 20 minutes post thermal inactivation onset.

When the luciferase of *P*. *leiognathi* undergoes thermal inactivation in *E*. *coli*, similar kinetics are observed at lower temperatures, which is explained by the presence of active bichaperone system DnaKJE-ClpB, which actively aids refolding [11]. However, as could be seen at Fig 2, in *B*. *subtilis dnaK*+ and *dnaK*-, kinetics of luciferase inactivation remain the same, while in *E*. *coli* the ratio of rate constants was at 1.8. This observation indicates that DnaKJE chaperone does not properly function in *B*. *subtilis*, as it is unable to support the refolding of luciferase.

**Fig 4:**
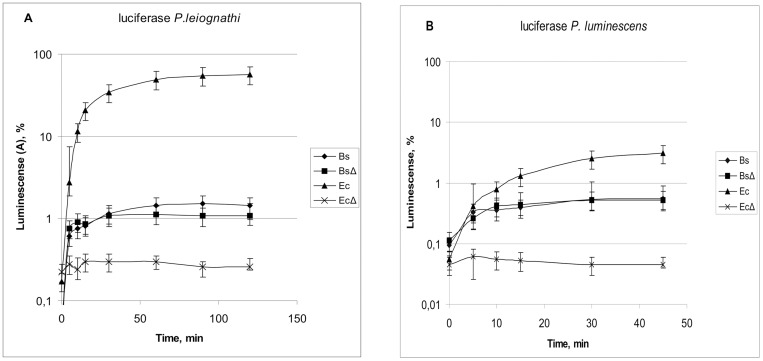
The kinetics of luminescence of *E*. *coli* and *B*. *subtilis* cells with thermal inactivated luciferases from *P*. *leiognathi* (A) and *P*. *luminescens* (B), which regained their activity after cell cultures were moved to room temperature. Luciferases were thermal inactivated *in vivo* by exposure of carrier cells at either 47°С (*E*. *coli)* or 51°С (*B*. *subtilis*). Relative luminescence shown on vertical axis is proportional to percent of refolded luciferase molecules. The cells of *B*. *subtilis* were pPfbaA-LeoAB (luciferase *P*. *leiognathi*) and pPfbaA-XenAB (*P*. *luminescens*), while the cells of *E*. *coli* were transformed with plasmids pTZ57R-LeoAB (*P*. *leiognathi*) and pTZ57R-XenAB (*P*. *luminescens*). Bs—*B*. *subtilis 168*. BsΔ—*B*. *subtilis* NBS2001 *ΔdnaKJ*. Ec—*E*. *coli* BW25113. *EcΔ—E*. *coli* JW0013 *ΔdnaK*::*kan*.

Fig 4 depicts the data describing the refolding of luciferases from *P*. *leiognathi* and *P*. *luminescens* after thermal inactivation *in vivo* in *E*. *coli* or *B*. *subtilis* cells. Bacterial cells were incubated at 47°С or 51°С for *E*. *coli* or *B*. *subtilis*, respectively. In both cases, the luminescence levels gradually decreased across approximately 2–3 orders of magnitude till reaching the background levels, at which point the translation inhibitors, chloramphenicol for *E*. *coli* and tetracycline for *B*. *subtilis*, were added to media followed by incubating bacterial cultures at room temperature under continuous monitoring of their luminescence. Thermal inactivation time was about 15–25 minutes.

As could be seen from the data presented on Fig 4A after thermal inactivation, the luminescence of *E*. *coli* cells expressing the *P*. *leiognathi* luciferase could be restored almost to its pre-inactivation levels. Evidently, this restoration is dependent on DnaK, as *E*. *coli* Δ*dnaK* cells are not able to restore their levels of luminescence after exposure to high temperatures.

In curves describing of the reactivation of thermally inactivated luciferases, both lag-period and inflection are unremarkable, thus, being indicative of possible multiples stages of reactivation reaction limited by some rate-limiting. Analysis of relatively fast refolding steps provides some difficulty due to rapid cooling down of the sample within the first few minutes after its return to the room temperature.

**Fig 5:**
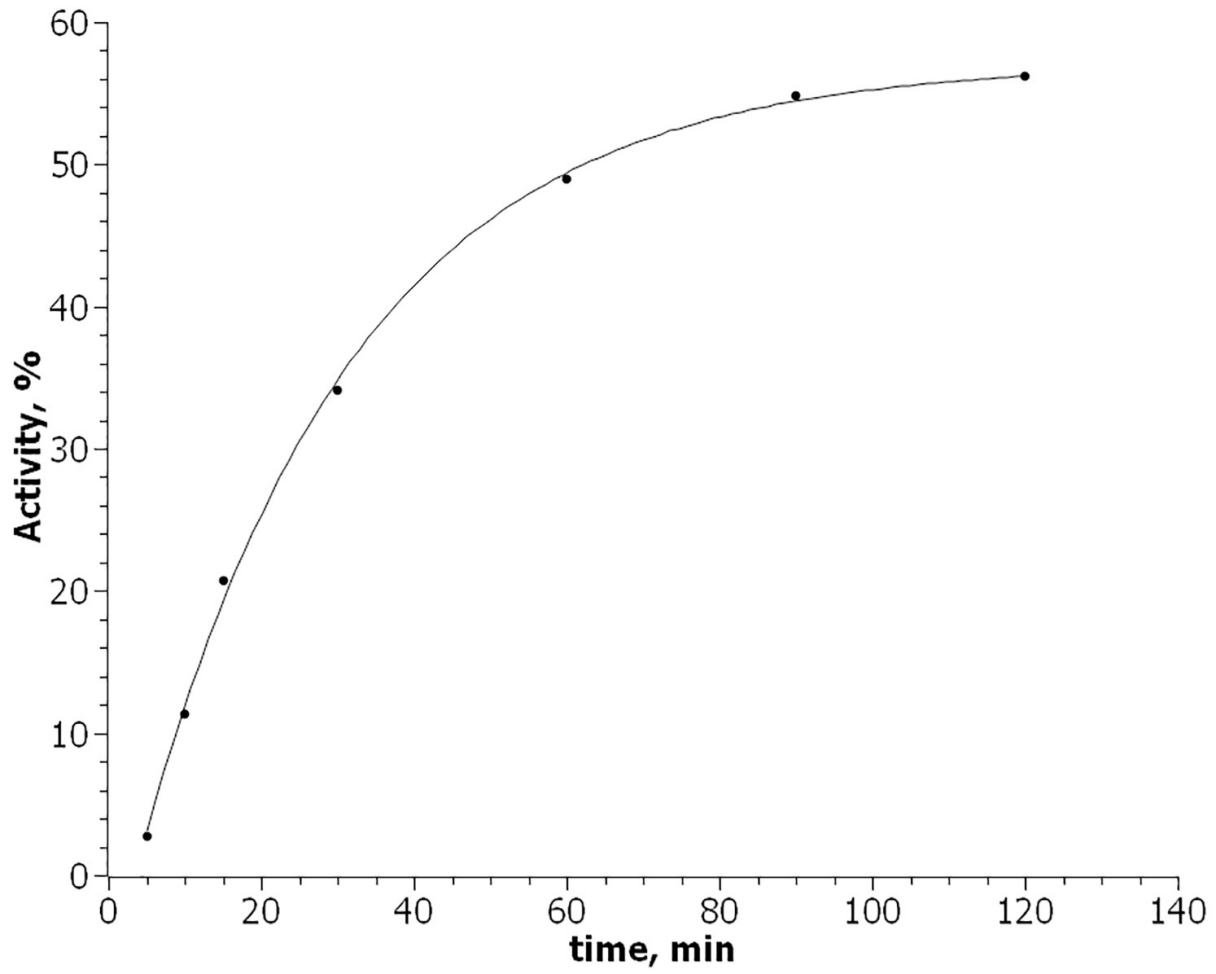
Non-linear approximation of a time-dependent increase of the activity of *P*. *leiognathi* luciferase in *E*. *coli* cells is described by first-order reaction kinetics with a lag-period.

Analysis of slower steps of *P*. *leiognathi* luciferase refolding in *E*. *coli* cells, most parts of the kinetic curve are well approximated by exponential dependence of an accumulation of the product resulting from the first-order reaction with lag-period A = A_max_×(1-e^k×(t-t*lag*)^), where A_max_ is the level of maximal degree of reactivation, k–first-order reaction rate constant and t*lag*–the time required for completion of fast steps of the refolding (Fig 5).

Kinetics curve parameters A_max_, k and t_lag_ were derived by non-linear approximation using SciDAVis software (A_max_ = 57,11±0,85%, k = 0,035±0,002 min^-1^ and t_lag_ = 3,42±0,44 min).

Refolding kinetics analysis of other strains showed that thermal inactivated cells of *B*. *subtilis* wild-type strain 168 are substantially less capable of luciferase refolding than *E*. *coli*, and successfully refold just approximately one percent of available denatured enzymes. In *B*. *subtilis*, the success of the refolding does not require the presence of DnaK chaperone. As opposed to *E*. *coli*, the cells of *B*. *subtilis* refold luciferases of *P*. *leiognathi* and *P*. *luminescens* to about the same levels, and with similar kinetics.

## Discussion

A comparison of thermal inactivation kinetics of luciferases in *E*.*coli* and *B*. *subtilis* strains transformed with lux-biosensor plasmids demonstrated that the thermostability of these model proteins in Gram positive bacteria *B*. *subtilis* is higher than that in Gram negative *E*. *coli*, with the difference in tolerated temperatures reaching 4–5°C (Figs 1 and 2).

Earlier works demonstrated that luciferase activity in *E*. *coli* cells depend on ability of particular luciferase to refold [10]. According to date presented above, in Gram positive bacteria *B*. *subtilis*, enhanced thermostability of bacterial luciferases is not because of better refolding. In fact, native *B*. *subtilis* cells do not support luciferase refolding well. After incubation of *B*. *subtilis* cells with luciferase-bearing constructs on elevated temperatures, bioluminescience drops 3–4 orders of magnitude; the transfer of these cells back to the room temperature results in restoring luciferase activity up to approximately 1% of its initial levels either in presence or in absence of *DnaKJ* chaperone. In conclusion, our experiments point that the role that DnaKJE plays in termostability of luciferases in *E*. *coli* is limited to this models system. In fact, in *B*. *subtilis* cells this chaperone is not involved in improving the thermostability of luciferase.

Possibly, activity of thermal inactivated luciferases in *B*. *subtilis* may be rescued by other ATP-dependent chaperones, which are yet to be investigated. A set of biosensors plasmids incorporating luciferases of varying intrinsic thermostability, which we presented above, may facilitate further molecular and genetic dissection of the factors which govern the denaturing and the refolding of recombinant proteins in Gram positive cells.

## Abbreviations

DnaKJE: DnaK DnaJ GrpE
RLU: relative light units
